# ﻿A new species of *Aleurolobus* Quaintance & Baker, 1914 (Hemiptera, Aleyrodidae) from China infesting *Murrayaexotica* L.

**DOI:** 10.3897/zookeys.1152.96447

**Published:** 2023-03-02

**Authors:** Qing-Song Lin, Lin-Qian Lu, Ji-Rui Wang

**Affiliations:** 1 College of Advanced Agricultural Sciences, Zhejiang Agriculture & Forestry University, Lin’an, Zhejiang 311300, China Zhejiang Agriculture & Forestry University Hangzhou China

**Keywords:** Aleyrodinae, morphology, new taxa, taxonomy

## Abstract

A new whitefly species, *Aleurolobusrutae***sp. nov.**, collected on *Murrayaexotica* (Sapindales, Rutaceae) leaves in the Maolan National Nature Reserve, Guizhou, China, is described and illustrated. Some of the individuals were infected with *Aschersoniaplacenta*, an entomopathogenic fungus. The insect is circular in shape and characterized by a very wide submarginal region, and the submarginal furrow is almost continuous, with only a small break at the caudal furrow. Anterior and posterior marginal setae are absent, but setae are present on the 8^th^ abdominal segment. Thoracic and caudal tracheal folds are discernible.

## ﻿Introduction

The whitefly genus *Aleurolobus* was erected by Quaintance and Baker (1914), with *Aleurodesmarlatti* Quaintance 1903, as its type species by original designation. The genus currently includes 90 species worldwide (Martin and Mound 2007; Dooley and Smith-Pardo 2013; Sundararaj and Vimala 2018); of these, only 16 species are known to occur in China (Dubey and Ko 2009; Yan and Bai 2017). The majority of species are from the Oriental Region. Dubey and Ko (2009) reviewed the species of this genus from Taiwan, China. *Aleurolobus* is recognized by having the submargin separated from the dorsal disc by a prominent furrow, the presence of eye spots, and the abdominal segment VIII forming a trilobed figure anterolateral to the vasiform orifice (Dubey and Ko 2009).

Recently, heavy infestations of a new whitefly, *Aleurolobusrutae* sp. nov., were discovered on *Murrayaexotica* L. trees in the Maolan National Nature Reserve, Guizhou Province, China. *Murrayaexotica* (Sapindales, Rutaceae) is an economically and medicinally important woody tree in China. It is widely used in daily life, especially in medical treatment, spices, seasonings, and other industries (Chen et al. 2020). It is one of the main crude materials of a patented Chinese compound drug “Sanjiu Weitai”, which is a traditional medicine for gastritis (Lu et al. 2021). The new species infesting *M.exotica* might affect the medicinal efficacy of the plant. We also found that some puparia were infected by *Aschersoniaplacenta*, an entomopathogenic fungus. Further studies of its biological characteristics are warranted to determine its potential as a biological control agent of the whitefly and its role in protecting this tree species.

## ﻿Materials and methods

Puparia of the new species were collected on *Murrayaexotica* trees in the Maolan National Nature Reserve, Guizhou, China; no adults were collected in the samples. The puparia were mounted following the method suggested by Dubey and David (2012). The terminology for morphological structures follows Bink-Moenen (1983), Martin (1985), and Gill (1990). The habitus images were taken using a digital camera Nikon D500 and Keyence VHX-6000 digital microscope from Guizhou University (**GZU**). Puparial measurements and microphotographs were taken using an Olympus (cx33) from Zhejiang Agriculture and Forestry University, Lin’an, China (**ZAFU**). The scanning electron microscope images were taken with a Hitachi SU8010 Scanning Electron Microscope (Hitachi, Japan) from Center of Electron Microscopy, ZAFU. Adobe Photoshop software was used to make small adjustments and to assemble the plates. The holotype is deposited in the Insect Collection of ZAFU.

## ﻿Taxonomic account

### ﻿ Aleurolobus rutae

Taxon classificationAnimaliaHemipteraAleyrodidae

Lin & Wang sp. nov.

B27FC458-75CB-5D88-87FE-DAA8A94A78B3

https://zoobank.org/EB4E769E-5F38-4819-B2B6-9C7851D0645E

#### Type material.

***Holotype*** puparium: China, Guizhou, Qiannan state, Maolan National Nature Reserve, 25°28.98'N, 108°07.10'E, 1190 m, 1 puparium on slide, 7. vii. 2022, leg. ST Meng, on *Murrayaexotica*, deposited in ZAFU, Lin’an, China.

**Figures 1–4.**
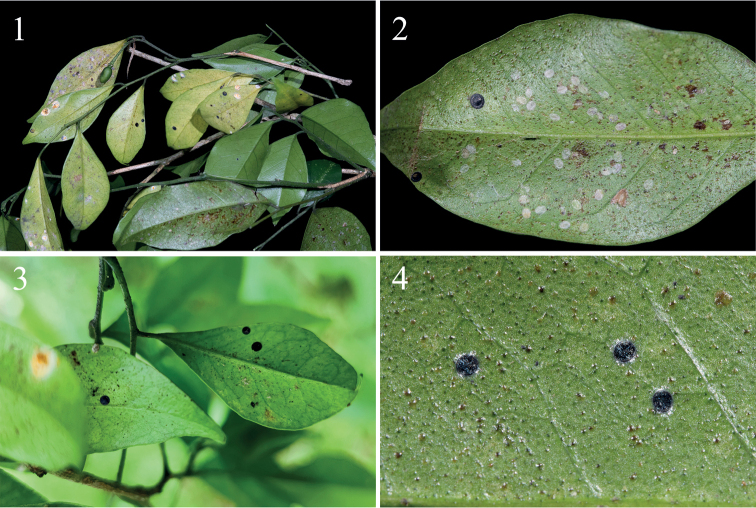
*Murrayaexotica* leaves infested by nymphs of *Aleurolobusrutae* sp. nov. **1, 3** puparia with some individuals infected with *Aschersoniaplacenta***2***Dialeuroporamurrayae* (pale nymphs) coexisting among *A.rutae* (dark nymphs) **4** third instar nymph of *A.rutae*.

***Paratypes***: 17 paratype puparia with same collection data as the holotype; of these14 puparia on 14 slides are deposited in ZAFU, 2 on 1 slide are deposited in Guizhou University and 1 on 1 slide are deposited in Shanghai Entomological Museum, Chinese Academy of Sciences.

#### Diagnosis.

**Figures 5–8.**
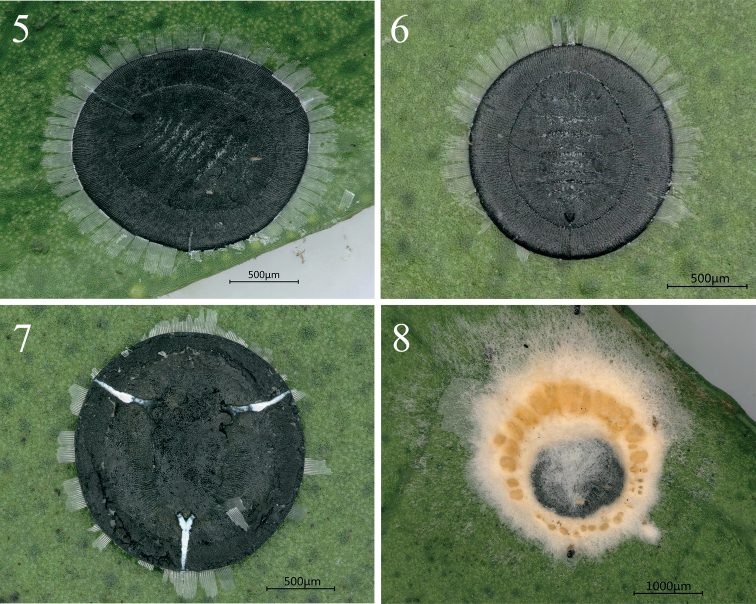
Live *Aleurolobusrutae* sp. nov. on *Murrayaexotica* leaves **5, 6** puparia in dorsal view **7** puparium in ventral view **8** puparia infected with *Aschersoniaplacenta* fungus.

**Figures 9–12.**
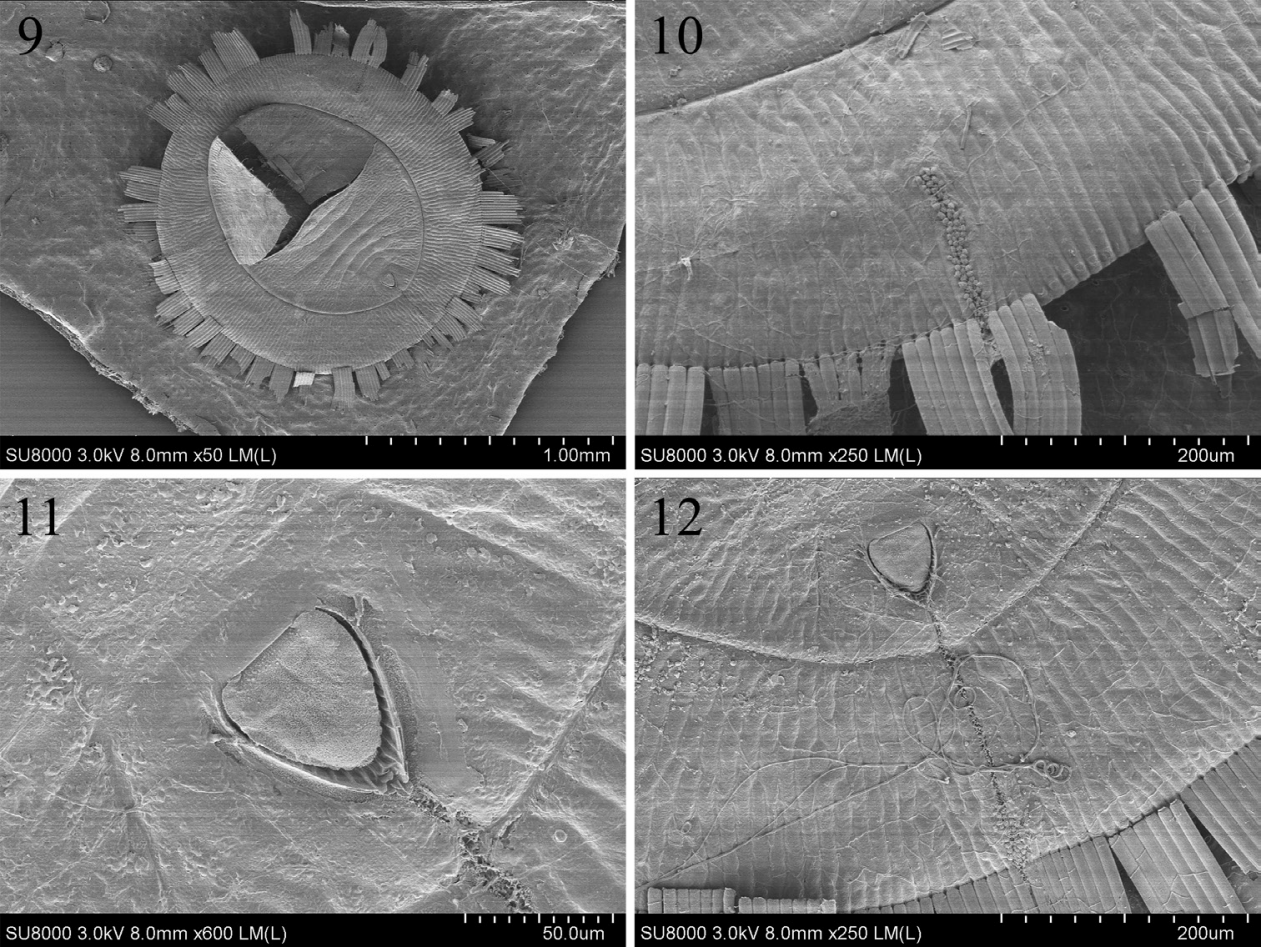
Scanning electron microscope photographs of *Aleurolobusrutae* sp. nov. **9** empty pupal case in dorsal view **10** thoracic tracheal fold and margin **11** vasiform orifice and operculum **12** caudal furrow.

**Figures 13–15.**
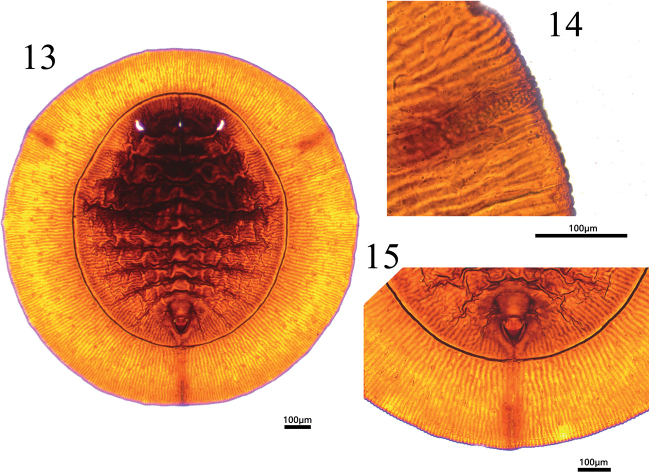
*Aleurolobusrutae* sp. nov., slide-mounted specimen **13** dorsum of puparium **14** lateral margin at tracheal opening **15** vasiform orifice, operculum, and caudal furrow.

**Figures 16–18.**
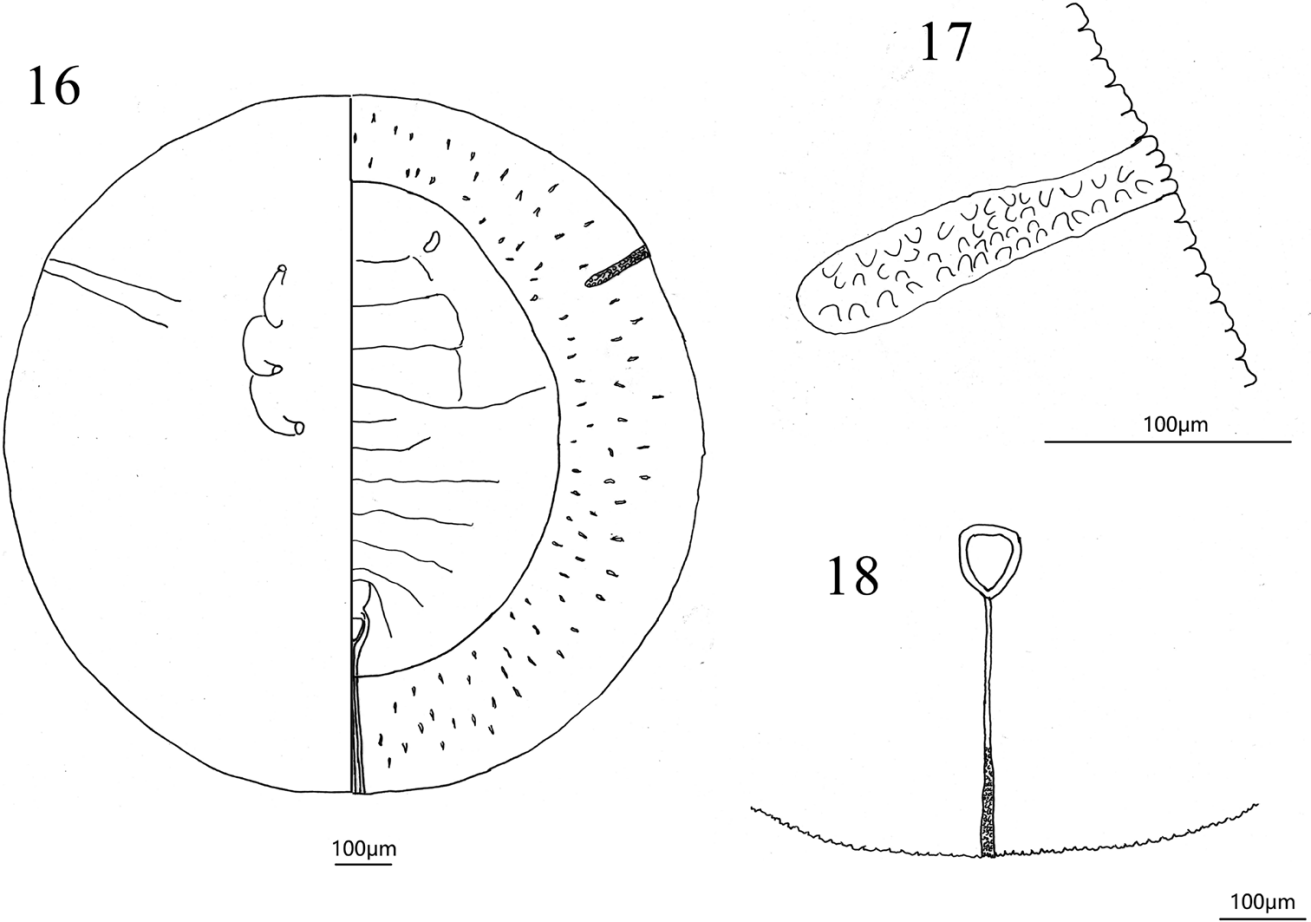
*Aleurolobusrutae* sp. nov., holotype puparium **16** puparium in dorsal (right) and ventral (left) views **17** margin **18** vasiform orifice.

*Aleurolobus* is recognized by having the submargin separated from the dorsal disc by a prominent furrow, the presence of eye spots, and the abdominal segment VIII forming a trilobed figure anterolateral to the vasiform orifice. The key characteristics that distinguish the new species from other *Aleurolobus* species is that it has a very wide submarginal region and lacks dorsal setae except the 8^th^ segment abdominal. Puparium black, circular, surrounded by a fringe of transparent shiny white wax and some wax deposition on the dorsum of the thoracic and abdominal segments, as well as along the thoracic tracheal folds (Figs 5–7, 9). The submargin is very wide and flattened and is separated from the dorsal disk by an uninterrupted submarginal furrow which extends around the entire body (Figs 5, 6, 9, 13, 16). An elongate, rectangular area with many minute tubercles extends from the tracheal opening approximately halfway to the submarginal furrow (Figs 10, 13, 16). The submargin in some specimens has 85–89 lanceolate setae present on each side, arranged in three rows (Figs 13, 15). The longitudinal molting suture and transverse molting suture both reach the submargin (Figs 9, 13, 16). The vasiform orifice is triangular, slightly longer than wide, lateral margins are rounded, with the basal ends curved to meet the basal margin; operculum triangular, almost covering the orifice and obscuring the lingual (Figs 11, 15, 18). Anterior and posterior marginal setae are absent. Caudal and dorsal setae, other than those in the submarginal region, are absent. The eighth abdominal segment setae is present (Fig. 11). Thoracic and caudal furrows are discernible (Figs 10, 12, 14, 15).

#### Description.

***Puparium*** black, large, 1.762–1.829 mm long, 1.725–1.833 mm wide, circular and nearly flat: the length–width ratio close to 1:1. Pupal margin surrounded by a fringe of transparent, shiny, white wax with some wax deposition on the dorsum of the thorax and abdominal segments, as well as along the thoracic tracheal fold (Figs 5–7, 9).

***Margin*** (Figs 10, 14, 17) crenulate, with eight or nine crenulations in 0.1 mm, each one with an apical notch. Anterior and posterior marginal setae are absent.

***Dorsum***: submargin broad and flat, separated from the dorsal disc by an uninterrupted submarginal furrow which extends around the entire body and with 85–89 submarginal, lanceolate setae present each side arranged in three rows (Figs 13, 15). An elongate, rectangular area with many minute tubercles extends from the tracheal opening approximately halfway to the submarginal furrow (Figs 10, 13, 16). The longitudinal molting suture and transverse molting suture both reach the submargin (Figs 13, 16). Thoracic and abdominal segment sutures are well defined; length of abdominal segments as measured along the midline as follows: abdominal segment I ~ 77.1 µm; abdominal segment II ~ 68.9 µm; abdominal segments III–V each ~ 81.2 µm; abdominal segment VI ~ 70.2 µm; and abdominal segment VII ~ 38.5 µm. Some small pores are present on dorsum.

Vasiform orifice (Figs 11, 15, 18) triangular, slightly longer than wide, 78.8 µm long, 69.9 µm wide; operculum triangular, 58.4 µm long, 55.4 µm wide, almost covering the orifice and obscuring the lingual. Vasiform orifice set anterior to the caudal end of the puparium by nearly four times its length. Caudal furrow 304.5 µm long. A pair of eighth abdominal setae present, ~10.0 µm, near the anterolateral margin of the vasiform orifice (Fig. 11).

***Venter***: thoracic and caudal tracheal folds discernible (Fig. 16). Ventral abdominal setae absent.

#### Third instar nymph

**(Fig. 4).** 0.82 mm long, 0.81 mm wide; the other morphological characteristics are basically identical with those of the puparium.

#### Host Plant.

*Murrayaexotica* (Sapindales, Rutaceae).

#### Distribution.

China (Guizhou).

#### Biology.

Three to five specimens were found per leaf (Figs 1, 3, 4), distributed on both sides of leaves, but especially on the upper side. This new species coexists with *Dialeuroporamurrayae* (Fig. 2). The puparium is covered by a thin layer of white wax, with highly characteristic secretions in the form of a broad, laterally directed, white fringe on each side of the body, 0.21–0.23 mm long (Figs 5, 6, 9). Some puparia were found infected with an entomopathogenic fungus (Figs 1, 8). Results of a polygenic sequencing analysis (ITS, *tef1- α*, SSU, LSU, RPB1, and RPB2; 100% similar to those in NCBI database respectively) identified the fungus as *Aschersoniaplacenta* Berk (Hypocreales, Clavicipitaceae), a highly effective pathogen of whitefly and scale insects (Wei et al. 2016). *Murrayaexotica* is an important medicinal plant, and the research and development of this fungal preparation may be helpful in reducing the damage of whitefly on this plant. The fungal specimen (No. ZHA-ZNL01) and its isolated strain (No. GZ-ZNL01) are both preserved in the Herbarium of Guizhou Institute of Technology. No ants were observed attending the whitefly.

#### Etymology.

The species is named for Rutaceae, the family of its host plant *M.exotica*. The specific epithet is a feminine genitive noun that does not change gender with respect to the genus.

#### Comments.

The puparium of the new species resembles that of *Aleurolobusrubus* in being round in shape, black in color, and in having a broad submargin. It differs in that the puparium of *A.rubus* is smaller and subcircular in shape. Additionally, the vasiform orifice is set anterior to the pupal caudal margin by four times of its own length in the new species, compared to that in *A.rubus*, which is twice its own length. The new species also resembles *Aleurolobusshiiae* but can be easily distinguished from that species which has an elongate, oval shape and minute tubercles located within the thoracic tracheal furrows that almost reache the submarginal furrow. The new species is also similar to *Aleurolobusolivinus*, but it has a very wide submarginal region, and the submarginal furrow is almost continuous with only a small break at the caudal furrow. *Aleurolobussubrotundus*, which has been found on the same host plant, has 10 pairs of long setae along the submargin, as compared to the absence of long setae in *A.rubus* and many minute, submarginal setae in *A.rutae*.

## Supplementary Material

XML Treatment for Aleurolobus rutae

## References

[B1] Bink-Moenen RM (1983) Revision of the African whiteflies (Aleyrodidae). Monografieën van de Nederlandse Entomologische Vereniging. Amsterdam 10: 1–211.

[B2] Chen CY Hou LY Lin B Zhan RT (2020) Study on genuineness and clinical application of Murrayae Folium et Cacumen. Liaoning Journal of Traditional Chinese Medicine 47(03): 161–164.

[B3] Dooley JW III Smith-Pardo A (2013) Two new species of whiteflies (Hemiptera: Sternorrhyncha: Aleyrodidae: Aleyrodinae) intercepted in quarantine on plants from Asia. The Pan-Pacific Entomologist 89(2): 84–101. 10.3956/2012-61.1

[B4] Dubey AK David BV (2012) Collection, preservation and preparation of specimens for taxonomic study of whiteflies (Hemiptera: Aleyrodidae). In: David BV (Ed.) The Whiteflies or Mealywing Bugs: Biology, Host Specificity and Management. Lambert Academic Publishing, Saarbrücken, 19 pp.

[B5] Dubey AK Ko CC (2009) A review of the genus *Aleurolobus* Quaintance and Baker (Hemiptera: Aleyrodidae) from Taiwan, based mainly on pupal morphology with a description of a new species. Entomological Science 12(1): 51–66. 10.1111/j.1479-8298.2009.00304.x

[B6] Gill RJ (1990) The morphology of whiteflies. In: Gerling D (Ed.) Whiteflies, their Bionomics, Pest Status and Management.Intercept, Andover, 13–46.

[B7] Lu MQ Liang HZ Tu PF Jiang Y (2021) Pharmacodynamic comparison of two different source plants of Murrayae Folium et Cacumen. Journal of Chinese Pharmaceutical Sciences 30(01): 49–57. 10.5246/jcps.2021.01.005

[B8] Martin JH (1985) The whitefly of New Guinea (Homoptera: Aleyrodidae). Bulletin of the British Museum (Natural History). Historical Series 50: 303–351. [Natural History]

[B9] Martin JH Mound LA (2007) An annotated check list of the world’s whiteflies (Insecta: Hemiptera: Aleyrodidae). Zootaxa 1492(1): 1–84. 10.11646/zootaxa.1492.1.1

[B10] Quaintance AL Baker AC (1914) Classification of the Aleyrodidae part II. Technical Series Burlin Entomology, United States 27: 97–105. 10.5962/bhl.title.123077

[B11] Sundararaj R Vimala D (2018) Description of two new aleyrodids of the genus *Aleurolobus* Quaintance & Baker (Hemiptera: Aleyrodidae) from India. Journal of Insect Biodiversity 7(2): 24–24. 10.12976/jib/2018.07.2.1

[B12] Wei XY Song XY Dong D Keyhani NO Yao LD Zang XY Dong LL Gu ZJ Fu DL Liu XZ Qiu JZ Guan X (2016) Efficient production of *Aschersoniaplacenta* protoplasts for transformation using optimization algorithms. Canadian Journal of Microbiology 62(7): 579–587. 10.1139/cjm-2015-0770 27192440

[B13] Yan FM Bai RE (2017) Whitefly Fauna of China. Henan Science and Technology Press, Zhengzhou, 59 pp.

